# A standardized *Hippophae* extract (SBL-1) counters neuronal tissue injuries and changes in neurotransmitters: implications in radiation protection

**DOI:** 10.1080/13880209.2017.1331365

**Published:** 2017-05-29

**Authors:** Madhu Bala, Vanita Gupta, Jagdish Prasad

**Affiliations:** Division of Radiation Biology, Institute of Nuclear Medicine and Allied Sciences, Brig. S K Mazumdar Marg, Delhi, INDIA

**Keywords:** Brain, ^60^Co-γ-irradiation, conditioned taste aversion, oxidative stress, plasma, sea buckthorn leaves

## Abstract

**Context:** Effects of a radioprotective, standardized leaf extract (code SBL-1) from traditional medicinal plant, sea buckthorn [*Hippophae rhamnoides* L. (Elaeagnaceae)], on neurotransmitters and brain injuries in rats showing radiation-induced conditioned taste aversion (CTA), are not known. Understanding CTA in rats is important because its process is considered parallel to nausea and vomiting in humans.

**Objective:** This study investigated the levels of neurotransmitters, antioxidant defences and histological changes in rats showing radiation CTA, and their modification by SBL-1.

**Materials and methods:** The inbred male Sprague-Dawley rats (age 65 days, weighing 190 ± 10 g) were used. Saccharin-preferring rats were selected using standard procedure and divided into groups. Group I (untreated control) was administered sterile water, group II was ^60^Co-γ-irradiated (2 Gy), and group III was administered SBL-1 before irradiation. Observations were recorded up to day 5.

**Results:** Irradiation (2 Gy) caused (i) non-recoverable CTA (≥ 64.7 ± 5.0%); (ii) degenerative changes in cerebral cortex, amygdala and hippocampus; (iii) increases in brain dopamine (DA, 63.4%), norepinephrine (NE, 157%), epinephrine (E, 233%), plasma NE (103%) and E (160%); and (iv) decreases in brain superoxide dismutase (67%), catalase (60%) and glutathione (51%). SBL-1 treatment (12 mg/kg body weight) 30 min before irradiation (i) countered brain injuries, (ii) reduced CTA (38.7 ± 3.0%, day 1) and (iii) normalized brain DA, NE, E, superoxide dismutase, catalase and CTA from day 3 onwards.

**Discussion and conclusion:** Radiation CTA was coupled with brain injuries, disturbances in neurotransmitters and antioxidant defences. SBL-1 pretreatment countered these disturbances, indicating neuroprotective action.

## Introduction

Sea buckthorn [*Hippophae rhamnoides* L. (Elaeagnaceae)] is a high altitude plant known for nutraceutical properties. In traditional medicinal systems, sea buckthorn has been used for the treatment of asthma, coronary heart diseases, gastric ulcers, hepatic injuries and skin diseases. Sea buckthorn leaves are valuable source of multiple bioactive compounds, such as flavonoids, tannins, triterpenes and vitamin C. Leaves are reported to be rich in α-tocopherol, plastochromanol-8, β-carotene, chlorophyll, quercetin 3-galactose, kaempferol, rutin and gallic acid (Guan et al. 2005; Kumar et al. 2011; Górnaś et al. 2014, 2016). The tea prepared from *Hippophae* leaves is popular for nutraceutical properties in China, Tibet and the Indian Himalayan region. A standardized and well-characterized leaf extract of *Hippophae rhamnoides* (coded as SBL-1, patent pending) demonstrated significant radiation protection in mice against cobalt-60 γ-irradiation (^60^Co-γ-irradiation). A single dose of SBL-1 [30 mg/kg body weight (b.w.)] administered 30 min before total body ^60^Co-γ-irradiation (TBI) at lethal dose 10 Gy, rendered survival of more than 90% mice population, whereas all of the non-SBL-1-treated irradiated (10 Gy) mice died within 14 days (Bala et al. 2009). Three marker compounds identified and quantified in per g SBL-1 were quercetin dihydrate (4.66 mg), rutin trihydrate (8.72 mg) and gallic acid ethyl ester (12.09 mg) (Bala & Saini 2013). Mechanistic studies showed that SBL-1 rendered radioprotection by multiple actions. SBL-1, when given before irradiation, countered radiation-induced oxidative stress, recombinogenesis and mutagenesis (Tiwari et al. 2009), prevented radiation-induced immunosuppression and inflammation by modifying the HMGB1-regulated pathway (Tiwari & Bala 2011), reduced radiation-induced micronuclei formation in bone marrow, histological changes and apoptosis in jejunum (Bala et al. 2015), and countered radiation-induced disturbances in the cellular populations of spleen (Bala & Kaur 2015). The radioprotective dose of SBL-1 countered the radiation-induced dysbiosis in jejunum microbiota (Bala et al. 2014) and promoted the tissue recovery in kidney (Saini et al. 2014). SBL-1 showed reversible binding to DNA (Saini et al. 2010).

The behavioural alterations induced after total body ^60^Co-γ-irradiation are still a very important concern. One of the important behavioural alteration termed as ‘conditioned taste aversion (CTA)’ studied in experimental rats after TBI (≤2 Gy) is a considered parallel process to nausea and vomiting in humans (Cairnie 1983). Radiation-induced CTA is often recommended for evaluating the performance of pharmaceuticals or drugs intended for use as radiation countermeasures (Cairnie & Leach 1982; Cairnie 1983; Shobi & Goel 2001). WR-2721, the only radiation countermeasure approved for clinical use with radiotherapy, was unable to counter the radiation-induced CTA in rats (Cairnie 1983). It was later found to be neurotoxic in humans (Czerwinski et al. 1972). To record the ‘radiation CTA’, the experimental rats are first conditioned to drink water only once in a day at a specified time; thereafter the solution of ‘preferred taste’ (e.g., saccharin) is introduced, which is followed by ‘the malaise’ (radiation in this case); and then the preference of animals towards ‘preferred taste’ (saccharin) after ‘the malaise’ (irradiation) is observed. Because of the very nature of this test, CTA is also viewed as a learning and memory task (Welzl et al. 2001). SBL-1 was demonstrated to counter the radiation-induced CTA in rats (Gupta et al. 2011).

Understanding the underlying mechanisms of radiation CTA is important for interpreting and evaluating the actions of radiation countermeasures. Gould and Yatvin (1973) reported the role of acetylcholine (ACh) in radiation CTA. Levy et al. (1974) showed that histamine was responsible for CTA. Subsequently, Rabin et al. (1982) showed that radiation-induced release of histamine had no role in acquisition of radiation CTA. Rabin and Hunt (1983) demonstrated that dopamine (DA) receptor antagonist (prochlorperazine) did not counter the acquisition of radiation CTA. Rabin et al. (1983) reported that ablation of area postrema (AP) of brain prevented radiation CTA in rats. Later, Rabin and Hunt (1986) reported the role of gastric distress and vagal afferent nerves in radiation CTA.

TBI to low linear energy transfer (LET) ionizing radiation causes injuries to multiple cells, tissues and organs by direct deposition of energy as well as by generating huge flux of free radicals, reactive oxygen species (ROS) and reactive nitrogen species leading to oxidative stress. The underlying mechanisms of radiation CTA are therefore expected to be multiple and complex. CTA in rats after TBI (2 Gy) was accompanied with an increase in the levels of serotonin (5-HT) in jejunum and plasma (Gupta et al. 2011). Also, it was demonstrated that radiation CTA was not countered by the neuronal disturbances in brain (Gupta & Bala 2014). The treatment with SBL-1 before irradiation countered radiation CTA by countering radiation-induced changes in 5-HT levels in jejunum and plasma (Gupta et al. 2011). Since the neurotransmitters are important determinants of a plethora of behavioural responses, the objective of the present study was to investigate whether radiation CTA was accompanied with changes in the levels of neurotransmitters in brain particularly DA, norepinephrine (NE), epinephrine (E), 5-HT, and in the levels of enzyme acetylcholinesterase (AChE). In addition, radiation-induced antioxidant defences as well as lesions in cerebral cortex, hippocampus and amygdala of rat brain were studied. Another objective of this study was to investigate whether SBL-1 treatment before irradiation had modifying effects on radiation-induced changes when given at doses, which countered radiation CTA in rats.

## Materials and methods

### Preparation of Hippophae leaves extract

The aqueous extract of *Hippophae* leaves (code name SBL-1) was prepared as per procedure described earlier (Bala et al. 2009). Briefly, the fresh green leaves of *Hippophae rhamnoides* grown in the Himalayas, identified by the botanist, Dr. O P Chaurasia of Defence Institute of High Altitude Research (DIHAR) as *H. rhamnoides* [specimen voucher No. SBTL-2006, DIHAR, Leh, India], were used. The leaves were collected in the month of June in year 2013; shade dried at 28 ± 2 °C, powdered and soaked in distilled water (100 g powder in 100 mL water for 3 days). The supernatant was lyophilized to obtain dried powder, which was coded as SBL-1. The HPTLC and HPLC profiles of SBL-1 were developed to match the marker compounds in SBL-1 prepared in different batches. The full chemical characteristics studied by HPTLC and HPLC of the batch of SBL-1 used in this study have already been published (Bala & Saini 2013; Bala et al. 2015, refer supplementary material).

### Experimental animals

**Figure 1.**
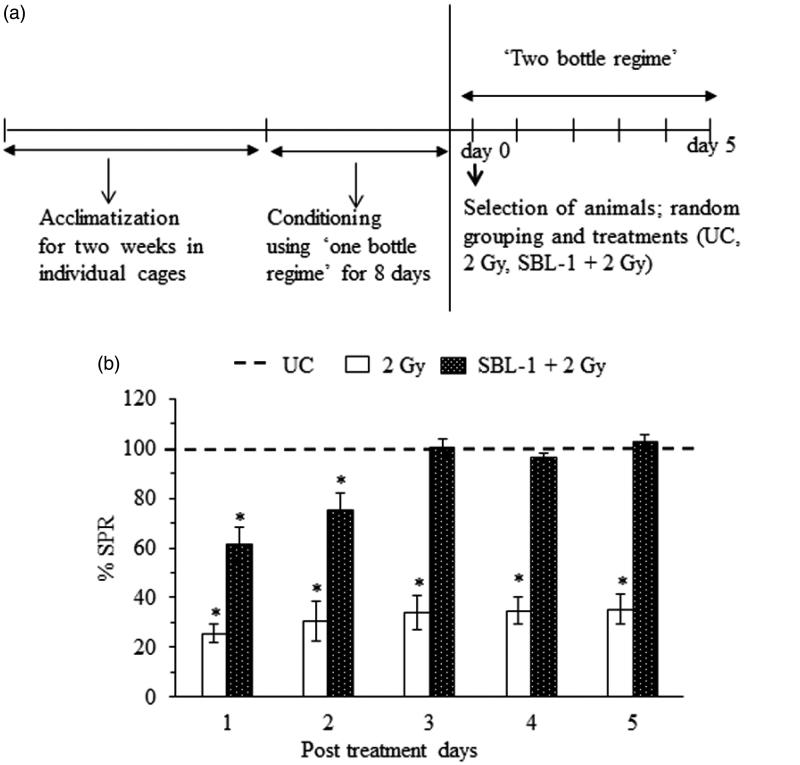
(a) After acclimatization in individual cages for two weeks, the rats were conditioned to take water only once a day for 30 min at a specified time on each day (water deprivation schedule of 23.5 h, ‘one bottle regime’). On day 0, the conditioned rats were selected on the basis of preference for intake of saccharin by using ‘two bottle regime’ where animals were given both water and saccharin simultaneously for 30 min only. Rats showing ≥50% intake of saccharin of the total fluid intake were selected. Selected rats were randomized and divided into three groups. Group I: untreated control (UC), group II: 2 Gy, group III: SBL-1 + 2 Gy. CTA was assessed in terms of saccharin preference ratio (SPR); {SPR (%) = [saccharin intake/(water intake + saccharin intake)] × 100}, decrease in SPR indicated acquisition of CTA. (b) It shows SPR (%) change in different groups. Data are presented as mean *±* SD of six rats after normalization with respect to UC. The SPR was 100% for UC. *statistically significant in comparison with UC at *p* < 0.05.

Inbred 65-day-old male Sprague-Dawley rats, weighing 190 ± 10 g, maintained under controlled environment at 25 ± 1 °C and 12h light–dark cycle in the Institute's Animal Experimental Facility, were used for the study. The rats were housed individually in polyvinyl cages, fed standard animal food pellets (Golden feed, Delhi) and were offered tap water *ad libitum* during acclimatization phase (Figure 1(a)). All experiments were conducted after approval of Animal Experimentation Ethics Committee of the Institute in accordance with the regulations specified by the Committee for the Purpose of Control and Supervision of Experiments on Animals (CPCSEA), India.

### Experimental design

#### Conditioning, selection and treatment of animals

For conditioning and selection of experimental rats, the standardized procedure was followed (Shobi & Goel 2001). Briefly, all the rats were subjected to water deprivation schedule of 23.5 h for 8 days. During these 8 days, each animal was offered tap water orally once a day for 30 min at a specified time (10:00 h–10:30 h) of the day (i.e., ‘one bottle regime’) and the intake of water by individual animal was recorded daily. On the 9th day (selection day, also referred as day 0 in Figure 1(a)), all the rats were given 0.1% saccharin solution and tap water simultaneously for 30 min (‘two bottle regime’). The intake of saccharin solution as well as tap water was recorded. The animals showing more than or equal to 50% intake of saccharin (0.1%) solution in comparison with water were selected for further experiments.

The selected rats were divided into three different groups: group I – administered sterile water only [untreated control (UC), *n* = 10]; group II – whole (total) body ^60^Co-γ-irradiated (2 Gy, *n* = 52); group III – administered SBL-1 30 min prior to whole body ^60^Co-γ-irradiation (SBL-1 + 2 Gy, *n* = 52), and *n* represents the number of animals.

For TBI, each rat was placed in a wire gauze container. 2 Gy radiation dose was delivered using Gammacell 220 (Atomic Energy Commission, Ottawa, Canada; dose rate of 0.23 rads/s) by varying exposure time. The SBL-1 was dissolved in sterile water, filtered and administered intraperitoneally (i.p.) as a single dose (12 mg/kg b.w.). The concentration of SBL-1 was 12 mg/kg b.w. because this concentration was earlier shown to counter radiation CTA effectively (Gupta et al. 2011). After every 24h interval, i.e., on day 1, day 2, day 3, day 4 and day 5, all the experimental animals were offered both the saccharin solution (0.1%) and tap water, simultaneously (‘two bottle regime’) for 30 min only. The intake of saccharin and water was recorded on each day. For determining CTA, the saccharin preference ratio (SPR) was calculated {SPR (%) = [saccharin intake/(water intake + saccharin intake)] × 100}. Decrease in SPR indicated CTA. The animals were sacrificed; brain and blood were collected for further studies.

### Preparation of brain homogenate and isolation of plasma

Animals were first anesthetized and then sacrificed humanely by cervical dislocation at 2 h, 5 h, day 1, day 2, day 3 and day 5. Six replicates (animals) were used to estimate the levels of neurotransmitters, AChE and antioxidant defences. Four replicates (animals) were used for histological studies. All animals of group I were sacrificed on day 1.

Whole brain was quickly dissected out and washed in ice-cold saline to remove the extraneous tissue. A 10% homogenate of brain was prepared in phosphate buffer saline (PBS, pH 7.2) using tissue homogenizer (Metrex Scientific Instruments Pvt. Ltd., Delhi, India). The tissue homogenate was used to estimate neurotransmitters (DA, NE, E and 5-HT), AChE, superoxide dismutase (SOD), catalase (CAT) and reduced glutathione (GSH).

The blood from heart was collected in tubes coated with ethylenediaminetetraacetic acid and plasma was separated from a part of blood. SOD, CAT and GSH were estimated in whole blood. The DA, NE and E were estimated in plasma. All the colorimetric detection and enzyme activity were done at 25 ± 1 °C using ELISA reader (Power Wave XS 2, BIOTEK, VT).

### Estimation of levels of neurotransmitters and AChE

Colorimetric detection of DA, NE and E was carried out using competitive 3-CAT Research ELISA kits as per the instructions provided by the manufacturer (LDN GmbH & Co. KG, Nordhorn, Germany). 5-HT was estimated using the competitive ELISA kits obtained from LDN GmbH & Co. KG. AChE (EC 3.1.1.7) was measured according to the method of Ellman et al. (1961). Extinction coefficient of thionitrobenzoic acid was taken as 1.36 × 10^4^ M^−1 ^cm^−1^. The AChE activity was normalized for protein concentration and expressed as AChE-specific activity (nmoles of thiocholine hydrolyzed per min per mg of protein).

### Assay for antioxidant defences

Methods of Marklund and Marklund (1974), Aebi (1974) and Beutler et al. (1963) were used to determine the activities of SOD (EC 1.15.1.1), CAT (EC 1.11.1.6) and the levels of GSH, respectively. Enzyme kinetics of SOD were recorded at 540 nm for 3 min (every 15 s) and change in absorbance/min was used to calculate the percentage of auto-oxidation inhibition to derive SOD units (U). CAT activity was assayed by recording change in absorbance at 240 nm for 150 s (every 15 s) and was calculated using extinction coefficient of 0.041 cm^2^ (μmol)^−1^. Bradford (1976) method was used to quantify protein.

### Histological studies

Whole brains were extracted carefully at day 1, day 2, day 3 and day 5 and frozen immediately in NEG 50 (cryostat frozen medium) at −20 °C. The 6-μ-thick coronal sections of brain were cut serially from the frozen blocks using cryostat (Leica Systems, Wetzlar, Germany) and mounted on glass slides. All the sections were stained with haematoxylin and eosin as per standard protocol. Microscopic observations were recorded by using light microscope (Axio Scope Observer D1, Carl Zeiss, Göttingen, Germany) at 50 × and 200×.

### Statistical analysis

Data for SPR were analyzed with ordinary two-way analysis of variance (ANOVA), followed by *post hoc* Bonferroni’s multiple comparison tests using software GraphPad Prism 6.0. Student’s *t*-test was used for comparing the two treatments. Data are presented as mean ± standard deviation (SD). *p <* 0.05 was considered as statistically significant.

## Results

### Radiation CTA and modification by SBL-1

In comparison with group I animals, group II animals showed significant CTA from day 1 to day 5 (Figure 1(b)). The radiation-induced CTA was countered significantly *(p* < 0.05*)* in group III animals on day 1 and day 2, and from day 3 onwards, no radiation CTA was observed (Figure 1(b)).

### Radiation-induced changes in the levels of neurotransmitters, AChE and their modification by SBL-1 treatment before irradiation

**Figure 2.**
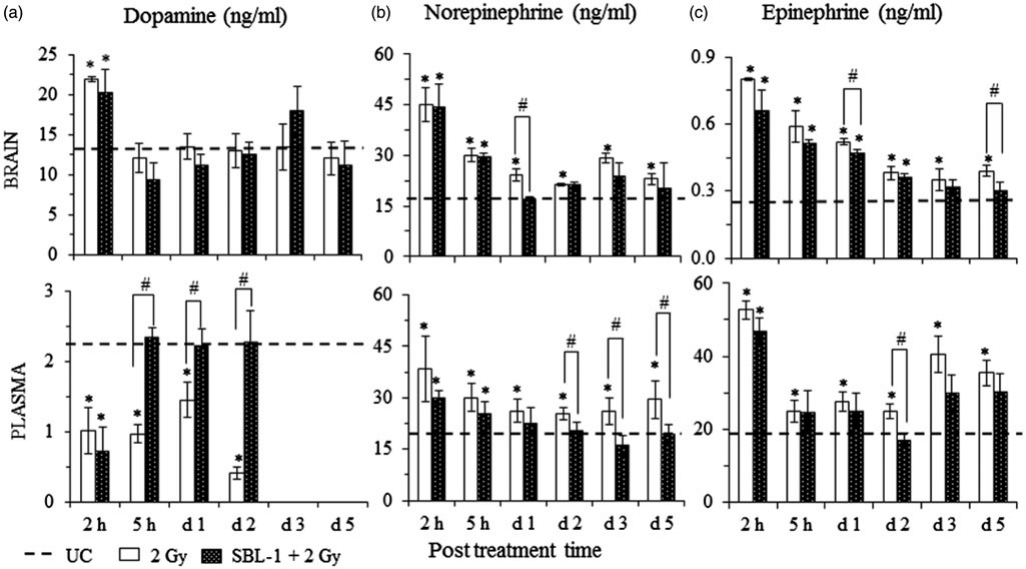
Modifying effect of *Hippophae* leaf extract (SBL-1) on radiation-induced changes in the levels of (a) dopamine, (b) norepinephrine and (c) epinephrine in brain and plasma of rats. Data are presented as mean ± SD of six rats in each group. *significantly different in comparison with untreated control (UC, group I) at *p* < 0.05 and ^#^significantly different in comparison with 2 Gy (group II) at *p* < 0.05.

In comparison with group I animals, the DA levels in brain of group II animals were significantly (*p* < 0.05) increased (63.4%) at 2 h only. The DA levels in plasma of group II animals were decreased significantly (*p* < 0.05) at all observation time points (Figure 2(a)). In group III animals (SBL-1 treated before irradiation), there was no change in the DA levels in brain in comparison with group II animals; however SBL-1 treatment before irradiation normalized the DA levels in plasma from 5 h onwards (Figure 2(a)).

The levels of NE in brain and plasma were increased significantly (*p* < 0.05) at all observation time points after ^60^Co-γ-irradiation (group II), in comparison with group I animals (Figure 2(b)). The increase was maximum at 2 h in brain (157%) as well as in plasma (103%). In group III animals, the normalized levels of NE were observed in brain and plasma from day 1 onwards (Figure 2(b)).

In comparison with group I animals, the group II animals showed significant (*p* < 0.05) increase in the levels of E in brain as well as in plasma at all observation time points (Figure 2(c)). The maximum increase in E was at 2 h and was 233% in brain, and 160% in plasma. Treatment with SBL-1 before irradiation (group III) normalized the levels of E in brain from day 3 onwards and in plasma from 5 h onwards (Figure 2(c)).

**Figure 3.**
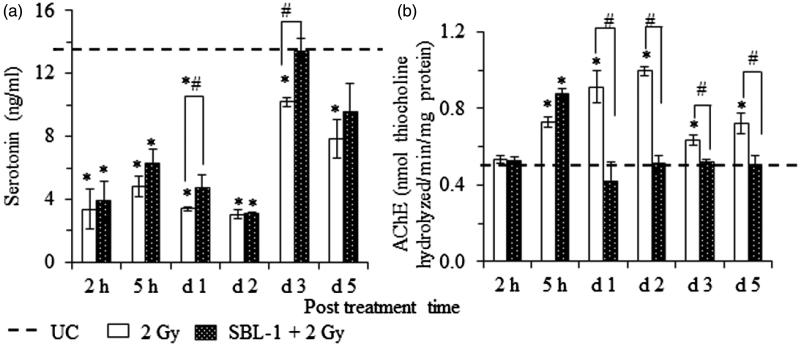
Radiation-induced changes and their modification by *Hippophae* leaf extract (SBL-1) on the levels of (a) serotonin and (b) acetylcholinesterase (AChE) in brain tissue of rats. Data are presented as mean ± SD of six rats in each group. *significantly different in comparison with untreated control (UC, group I) at *p* < 0.05 and ^#^significantly different in comparison with 2 Gy (group II) at *p* < 0.05.

In group II animals, the levels of 5-HT in brain showed significant (*p* < 0.05) decrease on all the observation days, in comparison with group I animals (Figure 3(a)). The levels of 5-HT in brain were higher in group III animals in comparison with group II animals, but the increase was significant (*p* < 0.05) on day 1 (39.2%) and day 3 (41.2%) only.

The AChE levels in brain of group II animals increased significantly (*p* < 0.05) from 5 h to day 5, in comparison with group I animals (Figure 3(b)). In group III animals, the radiation-induced increase in the AChE levels in brain was countered significantly (*p* < 0.05) from day 1 onwards and the levels were not significantly different than group I animals (Figure 3(b)).

### Effect of SBL-1 on radiation-induced changes in antioxidant defences

^60^Co-γ-irradiation (group II) significantly (*p* < 0.05) decreased the brain SOD levels at all observation time points (except at day 5), CAT and GSH levels at 2 h and 5 h, in comparison with group I animals (Table 1). In comparison with group I animals, there was no change in the levels of SOD and CAT in brain of group III animals, at all observation time points. In group III animals, the level of GSH was changed at day 3 only, in comparison with group I animals (Table 1).

**Table 1.** Effect of *Hippophae* leaf extract (SBL-1) on radiation-induced changes in the levels of superoxide dismutase (SOD), catalase (CAT) and reduced glutathione (GSH) in brain and blood of rats.

		SOD		CAT		GSH	
Time	Groups	Brain	Blood	Brain	Blood	Brain	Blood
	Group I	1.42 ± 0.08	10.5 ± 1.54	6.62 ± 2.04	153.6 ± 6.67	0.99 ± 0.14	3.30 ± 0.18
2 h	Group II	0.66 ± 0.04*	5.2 ± 0.53*	2.77 ± 0.49*	113.3 ± 12.2*	0.49 ± 0.03*	1.67 ± 0.05*
	Group III	0.83 ± 0.31	5.9 ± 0.96*	5.01 ± 0.34#	126.9 ± 13.2	0.55 ± 2.10	1.86 ± 0.22*
5 h	Group II	0.57 ± 0.01*	9.7 ± 2.52	2.68 ± 0.55*	106.2 ± 4.33*	0.59 ± 0.07*	2.00 ± 0.18*
	Group III	1.06 ± 0.32	10.6 ± 2.61	3.70 ± 0.45	140.6 ± 42.8	1.15 ± 0.01#	2.79 ± 0.1#*
day 1	Group II	0.71 ± 0.03*	10.1 ± 1.49	4.91 ± 1.37	116.4 ± 5.03*	0.81 ± 0.09	2.47 ± 0.49*
	Group III	1.36 ± 0.26#	13.8 ± 1.57#	7.74 ± 1.51	156.2 ± 5.61#	1.20 ± 0.14#	3.22 ± 0.13#
day 2	Group II	0.83 ± 0.09*	9.9 ± 3.07	6.09 ± 2.02	152.4 ± 5.48	0.93 ± 0.48	2.09 ± 0.35*
	Group III	1.44 ± 0.40	10.6 ± 1.79	9.57 ± 0.89	177.2 ± 12.3#	1.33 ± 0.23	2.31 ± 0.53
day 3	Group II	0.47 ± 0.14*	7.6 ± 1.26	6.86 ± 1.94	147.5 ± 15.3	1.10 ± 0.46	2.19 ± 0.43*
	Group III	1.76 ± 0.21#	10.3 ± 0.89#	12.41 ± 2.31#	192.8 ± 10.1*	2.22 ± 0.3#*	2.32 ± 0.52
day 5	Group II	1.08 ± 0.39	4.4 ± 1.91*	6.57 ± 1.72	145.8 ± 18.9	1.02 ± 0.06	2.52 ± 0.13*
	Group III	1.50 ± 0.11	10.5 ± 1.65	6.61 ± 1.03	179.1 ± 11.5	1.39 ± 0.14#	3.12 ± 0.32

Data are presented as mean ± SD of six rats in each group. Group I: untreated control (UC), Group II: 2 Gy and Group III: SBL-1 + 2 Gy.

* statistically significant in comparison with UC at *p* < 0.05.

# statistically significant in comparison with 2 Gy at *p* < 0.05.

GSH is expressed as nmol/mg protein; SOD as U/mg protein; CAT as μmol H_2_O_2_ consumed/min/mg protein.

In comparison with group I animals, group II animals significantly (*p* < 0.05) decreased the blood SOD levels at 2 h and day 5; CAT levels at 2 h, 5 h and day 1; and GSH levels at all observation time points (Table 1). In group III animals, normalized levels of SOD were observed from 5 h to day 5; normalized levels of CAT were observed at all observation time points (except at day 3); and normalized levels of GSH were observed from day 1 to day 5.

### Histological observations in rat's brain

**Figure 4.**
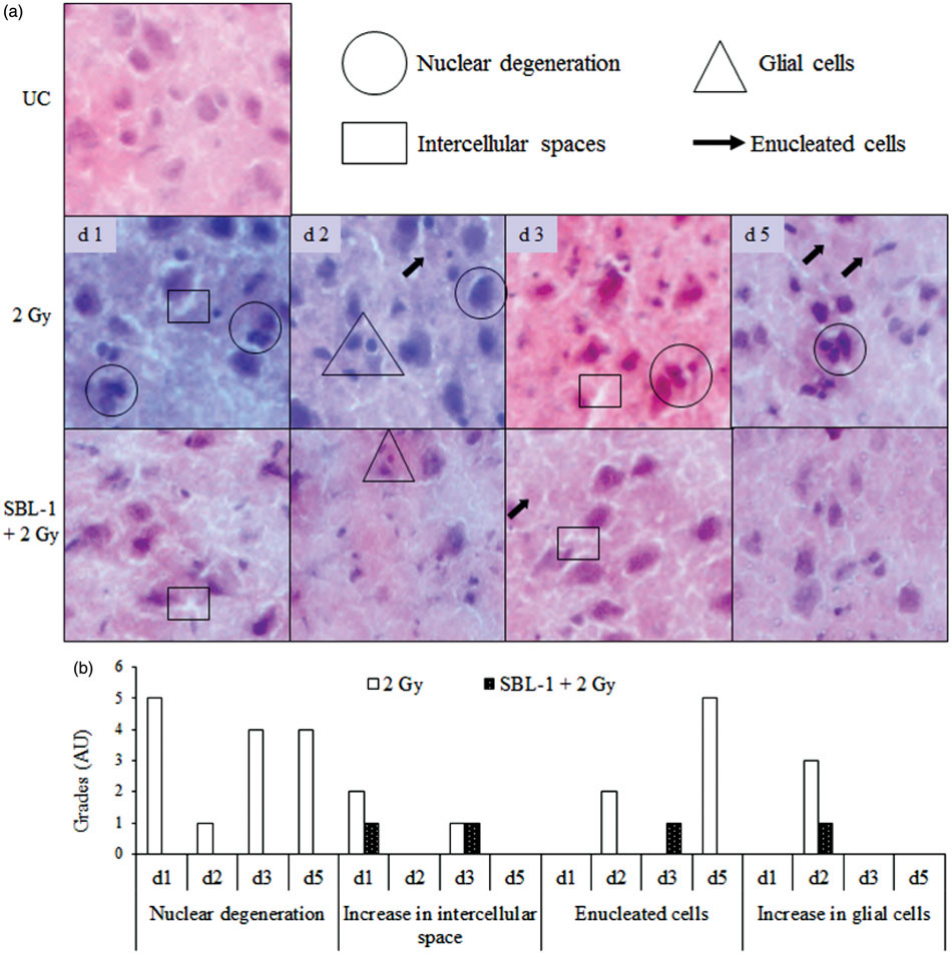
Effect of *Hippophae* leaf extract (SBL-1) on radiation-induced histological changes in cerebral cortex of rats. (a) Microscopic observations of haematoxylin- and eosin-stained tissue sections are presented at 200 × magnification. (b) Changes with respect to untreated control (UC, group I) are graded on number scale with 0 as minimum and 6 as maximum using arbitrary units (AU).

**Figure 5.**
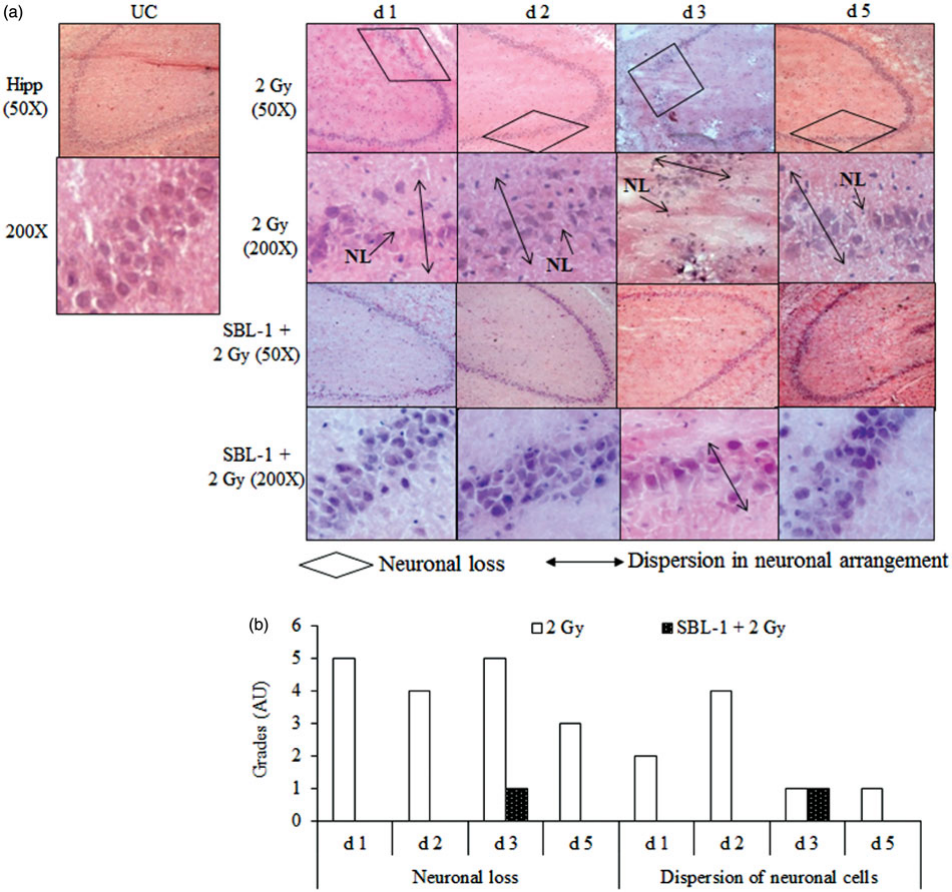
Effect of *Hippophae* leaf extract (SBL-1) on radiation-induced histological changes in hippocampus (Hipp) of rat brain. (a) Microscopic observations of haematoxylin- and eosin-stained tissue sections are presented at 50 × and 200 × magnifications. (b) Changes with respect to untreated control (UC, group I) are graded on number scale with 0 as minimum and 6 as maximum using arbitrary units (AU).

**Figure 6.**
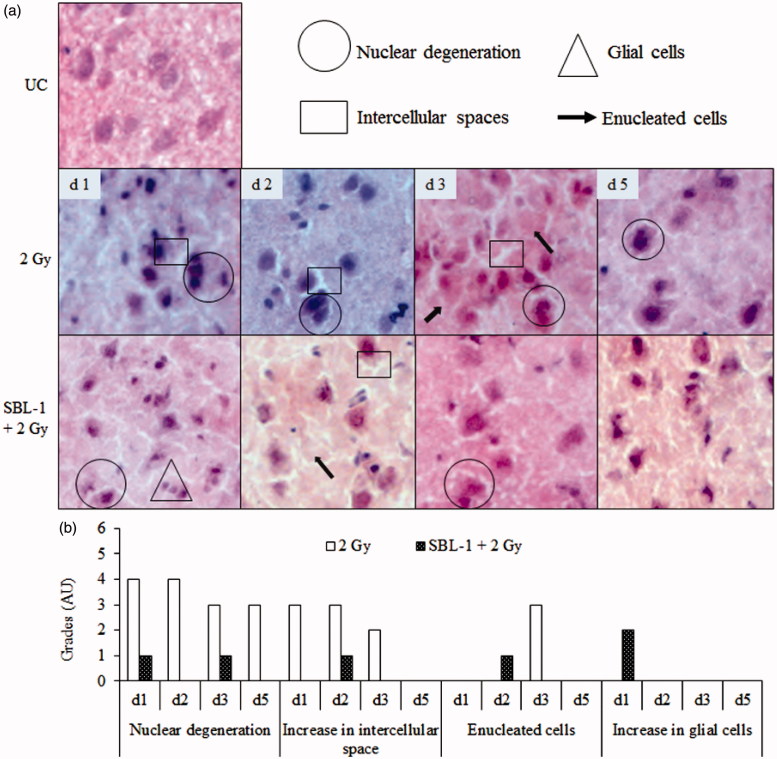
Effect of *Hippophae* leaf extract (SBL-1) on radiation-induced histological changes in amygdala of rats. (a) Microscopic observations of haematoxylin- and eosin-stained tissue sections are presented at 200 × magnification. (b) Changes with respect to untreated control (UC, group I) are graded on number scale with 0 as minimum and 6 as maximum using arbitrary units (AU).

Group I animals showed normal neuronal architecture in cortex, hippocampus and amygdala region of brain (Figures 4(a), 5(a) and 6(a)). The neuronal cells were compactly arranged, there were no nuclear degenerative changes, and clear demarcation of nuclear material from the cytoplasm was visible. Group II animals showed nuclear degeneration in cortex on all the observed days (Figure 4(a) and 4(b)). The intense and darkly stained nuclear material was observed in multiple cells of cortex which indicated degenerative changes in nuclei. In the same group, formation of intercellular spaces on day 1 and day 3, enucleated cells on day 2 and day 5, and increased glial cells on day 2 were observed in cortex. In group III animals, nuclear degeneration was prevented on all the observed days in comparison with group II animals. However, mild intercellular spaces were seen on day 1 and day 3, glial cells were visible on day 2 only, and small number of enucleated cells was seen on day 3 only. The tissue histology was normalized by day 5 in group III animals (Figures 4(a) and 4(b)). In group II animals, hippocampus region showed loss of neurons and dispersion of neurons on all the observed days, in comparison with group I animals (Figure 5(a) and 5(b)). In group III animals, no neuronal loss in hippocampus was observed up to day 2 while small neuronal loss and dispersion of neurons were seen on day 3, which was normalized on day 5 in comparison with group I animals. In group II animals, the amygdala region showed nuclear degeneration on all the observed days and formation of intercellular spaces on all the days except on day 5 and multiple enucleated cells on day 3, in comparison with group I animals (Figure 6(a) and 6(b)). In group III animals, mild nuclear degeneration was seen on day 1 and day 3. The observed changes were normalized by day 5, suggesting the recovery of lesions in the amygdala region, in the presence of SBL-1 before irradiation. In group III animals, the increase was observed in glial cells on day 1 in amygdala region.

## Discussion

CTA is a complex behavioural trait which involves learning, memory formation, consolidation of taste and visceral information, and retrieval of stored information (Welzl et al. 2001; Scott 2011). TBI causes a state of oxidative stress and damages multiple tissues. The presence of high amount of polyunsaturated fatty acids and high oxygen consumption makes the brain particularly susceptible to attack by oxidative species. The oxidation of cellular protein and nucleic acid leads to damage of neuronal cells, glial cells and membrane phospholipids. The inherent antioxidant enzymes, such as SOD and CAT as well as non-enzymatic antioxidant such as GSH, play important roles in countering oxidative stress (Sen & Chakraborty 2011). SOD dismutates the toxic superoxide radical into hydrogen peroxide, which is subsequently removed by CAT and/or glutathione peroxidase in the presence of GSH. GSH, a major non-protein thiol, plays an important role in the cellular antioxidant defence system. GSH scavenges free radicals, removes hydrogen and lipid peroxides, and prevents oxidation of biomolecules. In this study, the decrease in the levels of SOD, CAT and GSH at most observation time points in brain and blood in group II animals, indicated that the antioxidant defences were weakened after irradiation (Table 1). The neuronal injuries observed in cerebral cortex and amygdala (nuclear degeneration, increase in intercellular spaces and glial cells, and formation of enucleated cells) as well as in hippocampus (neuronal loss and dispersion of neuronal cells), within 24 h (day 1) in group II animals (Figures 4, 5 and 6), could be primarily due to oxidative damage caused by irradiation.

It was hypothesized that early lesions (at 24 h) in cerebral cortex, amygdala and hippocampus in group II animals (Figures 4, 5 and 6) should prevent the occurrence of CTA. This assumption was based on the published reports, where partial or complete ablation of specific brain areas interfered with the acquisition and retention of CTA (Scott 2011). Contrary to our assumption, the CTA was acquired within 24 h in group II animals which was retained up to day 5. Thereafter, it was argued that CTA could have occurred because of alterations in the levels of neurotransmitters in irradiated animals. Radiation plays an important role in damaging the neuronal tissues (Kempf et al. 2013). Changes in tissue histology and/or in the levels of neurotransmitters are reported in many disease models such as Parkinson, Alzheimer, Schizophrenia and Huntington as well as in many animal models (Bernert et al. 2003; Young 2009). The temporal changes in neurotransmitter levels were therefore measured in this study.

Since CTA is a response of many confounding functions, which involve emesis, pain perception, synaptic transmission, responses from environmental stimuli and regulation of bodily functions, the focus was on a study of related neurotransmitters (particularly DA, NE, E, 5-HT) and enzyme AChE. DA, a neurotransmitter of central nervous system (CNS) and peripheral nervous system (PNS), plays an important role in behaviour and cognition, reward, voluntary movement, motivation, punishment, working memory and learning. NE is a neurotransmitter of CNS and sympathetic nervous system. During synaptic transmission, NE is released from noradrenergic neurons. NE and E also act as stress hormones and are released from the adrenal medulla. NE prepares the brain to encounter and respond to stimuli from the environment and is responsible for vigilant concentration, whereas E is responsible for control of body tissues. 5-HT, present in CNS, PNS, platelets and enteric neural tissues, plays a very important role in pain perception, eating behaviour, learning, memory and thermoregulation. In comparison with UC, the significant increase in the levels of NE and E in brain as well as in plasma at all the time points after irradiation (Figure 2(b) and 2(c)), significant decrease in plasma DA from 2 h onwards (Figure 2(a)) and significant decrease in 5-HT levels in brain (Figure 3(a)) at all the time points in irradiated animals suggested the role of these neurotransmitters in radiation CTA (Figure 1). AChE is an enzyme, which is abundant in the synaptic cleft and hydrolyzes the free ACh (a neurotransmitter) into the inactive metabolites choline and acetate. Increased AChE levels from 5 h onwards after irradiation (Figure 3(b)) indicated the role of AChE in CTA. Immediate increase in brain DA levels at 2 h with a concomitant increase in NE and E levels at 2 h in brain as well as in plasma suggested that after irradiation, somehow, the conversion of brain DA to NE was triggered. The increased levels of NE in plasma may be due to their release from sympathetic nerves after irradiation. The increased levels of NE and E in brain as well as in plasma (Figure 2(b) and 2(c)) suggested that these neurotransmitters may have acted via activation of chemoreceptor trigger zone (CTZ) in the AP to cause CTA in rats. Reports showed that when NE and E were injected intracerebroventricularly, it produced emesis in cats. Ablation of the area postrema abolished the emesis in these cats (Beleslin & Strbac 1987; Jovanović-Mićić et al. 1989). The CTZ in the AP lacks the blood–brain barrier. The blood borne drugs or hormones send signals to CTZ and communicate with other structures in the vomiting centre to initiate vomiting.

In SBL-1 pretreated irradiated animals, normalization of DA, NE and E in brain and plasma (Figure 2) and AChE in brain (Figure 3(b)) at most observation time points indicated that normalization of radiation-induced changes in the levels of DA, NE, E and AChE was one of the important mechanisms by which SBL-1 countered radiation CTA (Figure 1). Countering the 5-HT levels in brain could have been another mechanism of action of SBL-1 (Figure 3(a)). The countering of radiation-induced increase in the levels of 5-HT in jejunum and in plasma by SBL-1 and its effect on CTA by activation of vomiting centres through 5-HT3 receptors present on the visceral afferent nerve endings was reported earlier (Gupta et al. 2011).

SBL-1 treatment before irradiation countered the radiation-induced changes in SOD, CAT and GSH (Table 1), indicating that the normalization of antioxidant defences was yet another mechanism by which SBL-1 countered radiation CTA (Figure 1). Earlier studies showed that SBL-1 treatment before irradiation countered the huge flux of radiation-induced ROS, free radicals (Tiwari et al. 2009; Saini et al. 2010), and was able to counter radiation-induced inflammation (Tiwari & Bala 2011) and derangement of tissue architecture of kidney (Saini et al. 2014) and gastrointestinal tract (Bala et al. 2015). Countering of radiation-induced oxidative stress by SBL-1 could be responsible for countering the neuronal injuries observed in cerebral cortex, hippocampus and amygdala (Figures 4, 5 and 6). The SBL-1 contained quercetin dihydrate (4.66 mg), rutin trihydrate (8.72 mg), gallic acid ethyl ester (12.09 mg), thiols (0.827 M total thiols), tannins (0.32 ± 0.006 g), proanthocyanidins (2.5% of total constituents of *Hippophae* leaves) (Bala et al. 2009; Tiwari et al. 2009; Bala & Saini 2013) and ellagic acid (Bala et al. 2015). These constituents are likely to be responsible for rendering strong antioxidant potential to SBL-1. Quercetin is an important flavonoid and is known to cross the blood–brain barrier (Youdim et al. 2004). It was reported to counter the neurotoxicity caused by 6-hydoxydopamine in rats by reducing oxidative damage in brain (Sriraksa et al. 2012). Proanthocyanidins are the oligomers of flavonoids, namely catechin, epicatechin and their gallic acid esters (epigallocatechin). Catechin and epicatechin are also reported to cross the blood–brain barrier (Faria et al. 2011). They have shown neuroprotective effects by decreasing oxidative stress and promoting spatial cognitive learning ability of rats (Haque et al. 2006). Dietary polyphenols have shown neuroprotection in patients of Alzheimer's and Parkinson's by attenuating oxidative stress and neuronal damage (Albarracin et al. 2012). Elseweidy et al. (2009) showed neuroprotective effect of green tea (catechins), which decreased the oxidative stress and neurotransmitter levels (NE, DA and 5-HT) in rats where diabetes was induced by streptozotocin. Some of the flavonoids like epigallocatechin 3-gallate and epicatechin provide neuroprotection not only by their antioxidant properties but also via activation of the various cellular signalling pathways, namely phosphorylation of extracellular signal-regulated protein kinase, phosphatidylinositol-3 kinase/protein kinase B and protein kinase C (Schroeter et al. 2007). Recently, gallic acid was reported to significantly protect the dopaminergic neurons and climbing abilities of *Dmp53*, *basket* and *drICE* gene knockdown fly lines (*Drosophila melanogaster*), which were treated with paraquat (oxidative stress generator) (Ortega-Arellano et al. 2013). Ellagic acid was reported to improve the 6-hydroxydopamine-induced Parkinson's disease in rats by reducing neuroinflammation and neuronal damage (Farbood et al. 2015).

## Conclusion

This study demonstrated that radiation-induced CTA in rats was coupled with disturbances in the levels of neurotransmitters, namely DA, NE and E in brain and plasma. Radiation CTA was also accompanied with reduced antioxidant defences and appearance of injuries to neural tissues in cerebral cortex, hippocampus and amygdala. SBL-1 treatment before irradiation countered radiation CTA, normalized enzymatic as well as non-enzymatic antioxidant defences, and countered most of the radiation-induced changes in the levels of neurotransmitters, namely DA, NE and E in plasma. It also countered radiation-induced changes in neuronal architecture and promoted the recovery of brain injuries.

This study together with our earlier studies suggests that SBL-1 is a promising candidate for developing a medical radiation countermeasure, which is an unmet demand globally.
